# Congenital rubella syndrome, a case series

**DOI:** 10.14745/ccdr.v50i78a05

**Published:** 2024-07-24

**Authors:** Olanrewaju Medu, Priyanka Mahajan, Maurice Hennink, Laurel Stang, Maureen Anderson, Ben Tan, Abimbola Oyenubi, Mireille Plamondon, Marina I Salvadori, Kristyn Franklin, Courtney Primeau, Joanne Hiebert, Jessica Minion, Tania Diener

**Affiliations:** 1 Saskatchewan Health Authority, Public Health and Preventive Medicine, Regina, SK; 2 Community Health and Epidemiology, University of Saskatchewan, Saskatoon, SK; 3 Population Health Branch, Saskatchewan Ministry of Health, Regina, SK; 4 Roy Romanow Provincial Laboratory, Regina, SK; 5 Department of Pediatrics, University of Saskatchewan, Saskatoon, SK; 6 Department of Pediatrics, University of Saskatchewan, Regina, SK; 7 Division of Pediatrics, Saskatchewan Health Authority, Saskatoon, SK; 8 Public Health Agency of Canada, Ottawa, ON; 9 Department of Pediatrics, McGill University, Montréal, QC; 10 National Microbiology Laboratory, Public Health Agency of Canada, Winnipeg, MB

**Keywords:** congenital rubella syndrome, Canada, immigration, public health management, risk mitigation

## Abstract

Rubella, or German measles, is a vaccine-preventable disease. Rubella infection is usually mild; however, infection in pregnancy is associated with severe outcomes for the baby, including pregnancy loss or a combination of developmental defects called congenital rubella syndrome. Within the last ten-year period, two cases of congenital rubella syndrome in Saskatchewan were reported to the provincial ministry and the Public Health Agency of Canada of the newborns of mothers who had recently arrived from Sub-Saharan Africa. Both infants had multiple health complications at birth consistent with congenital rubella and tested positive for the rubella virus. The article discusses the challenges encountered by the healthcare system in diagnosing, investigating, monitoring and managing cases of congenital rubella syndrome to prevent further sporadic transmission. The article emphasizes the need to provide additional support for cases and their households, especially new Canadians with less support to comply with public health advice and the importance of routine immunization to eliminate rubella globally.

## Introduction

Rubella, also known as German measles, is a vaccine-preventable disease of public health significance caused by the rubella virus. This disease typically presents in children and adults as a maculopapular rash, commonly preceded by a low-grade fever (1–3). While rubella infection is usually mild, infection in pregnancy is associated with severe outcomes for the baby, including pregnancy loss or a combination of developmental defects called congenital rubella syndrome (CRS). Congenital rubella syndrome can include low birth weight, heart, eye and hearing abnormalities, with or without microcephaly and other neurodevelopmental complications (2–4).

Prior to the introduction of rubella vaccines in the national immunization schedules, the disease would cause cyclic epidemics every three to 10 years (5). The last major rubella outbreak in the United States occurred from 1964 to 1965, causing 12.5 million infections, 20,000 cases of CRS, 11,000 pregnancy losses and approximately 2,000 neonatal deaths ((6,7)). In 2015, the Pan American Health Organization declared endemic rubella eliminated in the Americas, the first region to achieve this status (8,9). Rubella continues to transmit endemically globally, with prevalence highest in Africa, East Asia and South Asia (10).

The rubella vaccine was licensed in Canada in 1969. Soon after, the National Advisory Committee on Immunization endorsed a policy of mass immunization. Provinces readily initiated vaccination programs in the early 1970s, including Saskatchewan in 1971, leading to a significant decrease in the incidence of both rubella and CRS (9,11). The average incidence rate of rubella dropped from 37 cases per 100,000 people between 1969 and 1973 to fewer than one case per 100,000 people in 2005, the year rubella was eliminated in Canada (12,13). Since 2005, the rare cases of rubella or CRS diagnosed in Canada have been exclusively associated with virus importation (12).

Congenital rubella manifests either as congenital rubella infection or CRS. In congenital rubella infection, there is laboratory confirmation of infection in the absence of clinically compatible manifestations, while in CRS, there exists clinically compatible manifestations in addition to evidence of infection. The Canadian case definition requires the presence of any combination of the manifestations listed in **Table 1**.

**Table 1:** Congenital rubella syndrome clinically compatible manifestations

Column A	Column B
Cataracts or congenital glaucoma (either one or both count as one)	Purpura
Congenital heart defect	Hepatosplenomegaly
Sensorineural hearing loss	Microcephaly
Pigmentary retinopathy	Microphthalmia
	Developmental delay
	Meningoencephalitis
	Radiolucent bone disease
	Developmental or late-onset conditions such as diabetes and progressive panencephalitis and any other conditions possibly caused by rubella virus

Source: National case definition: Congenital rubella syndrome/infection

This article describes two recent cases of CRS in Canada, both acquired abroad. Given the relative rarity of these cases and lack of practical, updated guidelines regarding the public health management of CRS, we will also highlight public health management and risk mitigation approaches of sporadic rubella transmission.

## Case reports

### Case 1

The first case was born in the late 2010s from an immigrant mother, at a gestational age of 38 weeks and four days. At delivery, the baby was observed as small for gestational age, weighing 2.33 kg (i.e., less than the third percentile). The Apgar scores at birth were four and eight at one and five minutes, respectively. Clinical assessment of the infant in the immediate post-partum period revealed neonatal jaundice with an elevated total bilirubin of 307 µmol/L on admission to the neonatal intensive care unit. Further assessment revealed evidence of radiolucent bone disease, thrombocytopenia of 70 × 10^9^ platelets/L and a patent ductus arteriosus.

Nasopharyngeal and throat swabs taken on day 25 after birth were positive for rubella virus by real-time reverse transcriptase-polymerase chain reaction (RT-PCR). A urine specimen was negative. Testing for inborn errors of metabolism was negative.

Upon review of the case, the mother and the family were found to have immigrated from Sub-Saharan Africa to Regina, Saskatchewan earlier in the calendar year. At the time, the mother was at approximately 12 weeks of gestation, but unaware of the pregnancy.

At the initial prenatal visit, the mother did not present written records of immunizations from her home country; however, she stated she was immunized as a child. Her serology showed a high rubella IgG titre, greater than 500 IU/ml. Titres for IgM were not performed, as the high-rubella titre values were thought to reflect immunity. The pregnancy was unremarkable and the mother did not recall any rash or flu-like illness. It is important to note that asymptomatic and subclinical rubella presentations do occur. Furthermore, the national immunization schedule in this patient’s home country did not include rubella.

Over the next five years, the child was monitored by a paediatrician and developed a number of adverse sequelae consistent with congenital syndrome.

### Case 2

A few years later, the public health office was alerted to a positive IgM rubella result in a two-day-old neonate, which was confirmed at the National Microbiology Laboratory by two additional independent methods. In this case, the mother had arrived in Regina, Saskatchewan from Sub-Saharan Africa at 29 weeks gestation. The baby was born to a 30-year-old mother via urgent caesarian section at 38 weeks and four days of gestation. Apgar scores at birth were four and eight at one and five minutes, respectively. The paediatrician noted microcephaly, with a head circumference of 31.5 cm (less than the third percentile) and dry, scaly, peeling skin. The rest of the newborn exam was unremarkable. A nasopharyngeal swab collected seven days after birth was positive for rubella virus by real-time RT-PCR (14). The urine specimen collected at the same time was inconclusive.

A case review revealed that an initial prenatal visit at 30 weeks showed an elevated rubella IgG titre of 256.6 IU/ml. As with the previous case, while the mother reported being vaccinated as a child, there were no written immunization records and the national immunization schedule of the country of origin did not include rubella vaccine.

Radiolucent bone disease was confirmed on X-rays done at two weeks of age and at the time of the writing of this report. Additional investigations are ongoing. Echocardiography showed a normal cardiac anatomy.

## Public health management

This section highlights the public health response and the measures taken to mitigate the transmission risk from these cases.

### Case notification and investigation

**Figure 1:**
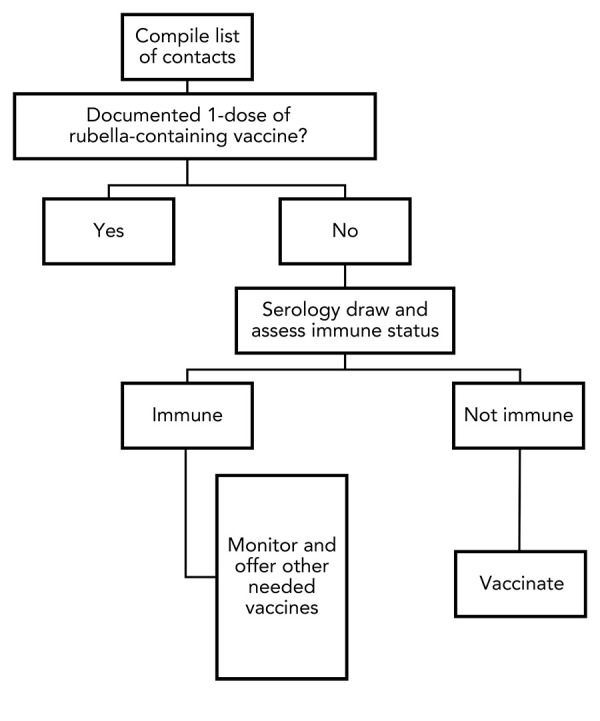
Flowchart to determine public health action based on immunity

Following notification of the cases to local public health, investigations were initiated into the chronology of events to identify any possibility of in-country disease acquisition, as part of a risk assessment (**Figure 1**). Given the mothers’ dates of arrival, dates of delivery and clinical histories, it was concluded that their rubella infections were unlikely to have been acquired within Canada and that only the infants were considered infectious.

## Transmission risk infection control

Children with CRS are considered to be infectious for the first year of life, unless repeated pharyngeal and urine testing (by RT-PCR and/or viral culture) are negative. In the home context, the risk is limited to non-immune contacts (especially susceptible pregnant visitors). Lessons learned from managing the first case were applied in the investigation and management of the second.

### Dedicated staffing

As new immigrants are often less familiar with the Canadian healthcare system, dedicated staff were assigned to interface with the patients and their families. This helped to build trust and ensure consistency of practice, but also served to mitigate occupational risk exposures by limiting the number of people exposed to the case to a small number of professionals with confirmed rubella immunity.

### Contact identification and management

To prevent further transmission within Canada, the focus was on the cases; immediate families, close contacts, healthcare contacts and the larger community.

The rubella immune status of immediate familial contacts was assessed. The National Advisory Committee on Immunization currently recommends a single dose of rubella vaccination to be considered immune, while two doses are required for immunity against measles, mumps and varicella (14). When no documented immunity or record of a rubella vaccine dose was available, a dose of rubella-containing vaccines (measles-mumps-rubella, or MMR) was provided. Where the measles vaccination record was also lacking, we offered a second dose of MMR to provide two-dose protection against measles. Additionally, for contacts with documented immunity but no documented evidence of receiving rubella vaccines, a similar catch-up schedule was offered, including rubella-containing vaccines (**Table 2**) (15).

**Table 2:** Summary table of public health actions

Individual/group	Public health actions
Mother	Had proof of immunity to rubella presumably from natural disease and offered catch-up vaccination
Newborn	Conducted ongoing clinical management for sequelae of CRS Practiced contact and droplet precautions while infectious Collected monthly nasopharyngeal swabs and urine samples for rubella RT-PCR
Household contacts	Verified rubella immunity status and offered catch-up vaccination
Healthcare providers	Practiced contact and droplet precautions for all encounters with the CRS case, as long as infectious Verified rubella immunity of exposed and potentially exposed healthcare provider(s)
Visitors to hospital and home	Monitored for symptoms among unvaccinated visitors prior to the case diagnosis until the end of the rubella incubation period; i.e., 12 to 23 days Subsequently restricted visitation to persons with documented immunity, with the appropriate contact and droplet precautions

Abbreviations: CRS, congenital rubella syndrome; RT-PCR, real-time reverse transcriptase-polymerase chain reaction

As these were new Canadians who often rely on support from persons from their country of origin (who might similarly not have rubella vaccination), lists of non-familial contacts were requested, assessed for immunity and provided vaccines where necessary. Recommendations were also provided that future contacts until the baby is no longer infectious be limited to rubella-immune individuals.

### Social events

Cultural and religious events have significance for this population and we worked to ensure that these events were modified to limit the risk of transmission. Some of the activities identified include baby naming ceremonies, religious events and commemorative feasts. Modifications included smaller group sizes and only rubella-immune persons in attendance.

### Healthcare-related infection control and environmental hygiene

The family was advised to maintain contact and droplet precautions, as well as ensuring a two-metre distance between baby and unimmunized persons where possible. Given that transmission is through nasopharyngeal secretions and urine, guidance was provided on dealing with potentially contaminated items.

With the expected increased frequency of healthcare visits, exposures in these settings were anticipated and the risk of transmission mitigated by appropriate infection control precautions. In the acute care component of the healthcare system, the patients were flagged using a similar process used for other medically important infections on the arrival electronic system. The mother-baby pair was flagged because the baby in most cases accompanied the mother. In addition, we proactively communicated with the facilities where visits were expected regarding infection control practices to mitigate exposure. We replicated this proactive communication with independent physician offices and the sample letter provided is shown in the **Appendix**.

Furthermore, clinical offices were advised to limit the pool of staff and patients with potential for contact with the infant. Similar to the measures taken by public health staff, documented proof of immunity was required for staff who provided care to the patient (Appendix).

Rubella is an enveloped virus, which makes it susceptible to the cleaning products used for low-level disinfection (2). Safety data sheet information notes that the virions are susceptible to either chloroform, formaldehyde, 1% sodium hypochlorite and 70% ethanol-based disinfectants (16). In practice, regular environmental cleaning supplies such as accelerated hydrogen peroxide wipes would provide sufficient disinfection. It is best to use products commonly used for disinfection in hospitals and households on a regular basis.

### Laboratory testing

The public health staff conducted monthly home visits to assess the infant and collect nasopharyngeal swabs and a urine sample for rubella RT-PCR. Given the expected prolonged viral shedding in babies with CRS, two consecutive negative RT-PCR results one month apart would be required to medically clear the baby and conclude that the infant is no longer infectious, which is the same period required by the Pan American Health Organization to achieve adequate CRS surveillance in an elimination setting (17–20). Ultimately, we medically cleared our first case in the twelfth month of life.

For both CRS cases, both a nasopharyngeal swab and urine specimen were collected early after birth and only the nasopharyngeal swabs were RT-PCR positive. In the first case, the nasopharyngeal swab became negative at eight months of age. The urine became RT-PCR positive at three months of age and remained positive longer than the nasopharyngeal swab, still being positive at 10 months of age. For the second case, the urine returned a first negative at three months of age while the nasopharyngeal swab remained positive as of four months following the first swab. The laboratory manual developed by the World Health Organization (WHO)’s Global Measles and Rubella Laboratory Network notes that throat swabs are the preferred specimen for CRS confirmation by RT-PCR (21). The reduced sensitivity for rubella viral detection by RT-PCR seen in the urine specimen is likely related to the difficulty in obtaining urine specimens in adequate volume (more than 10 ml) from infants.

The use of RT-PCR testing could lead to a longer isolation period compared to that for a viral culture (22,23). This is because a viral culture detects only infectious virus, while RT-PCR can also detect neutralized or inactive virus.

Using WHO’s standardized rubella genotyping methods, the first case was determined to be of genotype 2B and the second case of genotype 1G (24,25). Genotype 2B has a wide global distribution and has been noted to be endemic in African countries near the mother’s country of origin (26). On the other hand, relatively few sequences of genotype 1G have been reported and none have been reported to the WHO Global Rubella sequence database in recent years (27). The detection of this imported 1G case likely reflects a lack of sufficient genotypic surveillance in areas with higher rubella virus circulation and highlights the importance of obtaining rubella genotypes in cases occurring in low prevalence rubella countries such as Canada.

### Travel, transit, housing and other considerations

Both cases were new Canadians, with relative unfamiliarity with local services. We identified multiple challenges including transportation, housing, immigration documentation and childcare needs.

Our public health team provided transportation support to and from medical appointments until the infants were deemed no longer infectious. For other non-medical transportation within the city, our recommendation was the use of personal vehicles where available and possible, followed by single passenger transportation modes and finally public transit if needed. We provided guidance about contact and droplet precautions and this, in our assessment, mitigated transmission risks on public transportation. We recommended against air travel given the hypothetical infection risk to unvaccinated pregnant contacts during travel.

For childcare, we were explicit in informing the parents/caregivers of both cases that the cases could not attend regular daycares due to the possible presence of non-immune persons. Instead, public health encouraged the parents to identify immune persons who were able to provide needed childcare.

Housing and immigration documentation needs were outside of the mandate of the public health teams; however, we established connections with the respective agencies, both provincial and federal, and advocated on behalf of our clients with varying levels of success.

### Case reporting

Due to the rare nature of CRS in Canada, these case reports were highly scrutinized by provincial and national health agencies, requiring that local public health provide extensive case investigation details. Furthermore, Canada committed to the Pan American Health Organization’s goal of rubella elimination in the Americas in 2005 and as part of Canada’s commitment to the International Health Regulations, all cases of rubella must be reported to the WHO ((12)). These case reporting requirements are important to maintain global health security and contribute to global guidance on the management of public health communicable disease risks.

## Public health learning points

Over the course of responding to and managing these cases, we identified several learning points.

First, a positive rubella IgG is generally assumed to represent vaccine-derived immunity in Canadian-born pregnant women. However, this should not be assumed for persons arriving from countries where rubella activity is still ongoing. In both of the cases described in this article, the mothers had significantly elevated rubella IgG titres, which were interpreted as reassurance of immunity when they would have benefitted from additional evaluation. Consequently, we suggest that during pregnancies in which positive rubella IgG with elevated titres is seen, clinicians consider requesting a rubella IgM test in pregnant women whose childhood immunization history is unclear and who have recently been in areas with endemic rubella (23). Rubella IgG avidity testing can further be used to differentiate a recent exposure (low avidity) from a past exposure (high avidity) (23,28).

Secondly, due to the reduced incidence of rubella disease in Canada, there is a limited pool of experience to inform the best public health management and surveillance practices (18,29–31). As with most public health questions, we ultimately balanced the benefits of our risk control and mitigation approach with any harms that may occur. As far as we are aware, no further transmission occurred from the first case. Therefore, it would be helpful to have national guidelines to inform the public health management of a case of congenital rubella infection and CRS.

Finally, it is important to note that recent immigrants may face additional challenges when complying with public health advice, due to their limited ties to the community and access to supportive resources. As such, it may be necessary to provide additional assistance to cases of congenital rubella infection and CRS to ensure they have the resources required to comply with recommendations to mitigate the spread of illness. We recommend that jurisdictions consider providing resources to support these cases until they are no longer contagious.

## Conclusion

International travel in an increasingly global community impacts disease transmission dynamics and facilitates incident cases of imported communicable disease from high-incidence to low-incidence jurisdictions. It is expected that this will only increase as the impacts of the COVID-19 pandemic slowly come to light, including decreased routine childhood vaccine uptake coupled with “pent up” international travel demands (32). This emphasizes the critical importance of preventing and controlling vaccine-preventable diseases in both of these contexts.

As illustrated in these cases, not all countries offer childhood rubella immunization, which continues to impact global ability to eliminate this infection. Rubella elimination is achievable with routine immunization at the population-level. The WHO should be supported to facilitate the Expanded Programme on Immunization where it is needed most, in countries with high incidences of preventable conditions.

We describe the intensive resources at the local level required to manage two cases of CRS in a low-incidence, high-income country setting. Regardless of the immediate public health follow-up of infectious cases, these cases emphasize the very real risk of lifelong adverse outcomes among babies born with CRS. Our priority was ensuring sporadic transmission in Canada did not occur; however, the international public health community as a whole should be equally concerned with preventing and eliminating rubella globally. Rubella-containing vaccines have high efficacy, immunogenicity and safety; all children should have the opportunity to be immunized.

## Appendix Sample Letter to healthcare practitioners

Dear Provider,

Re: XXXXXXXXXXXXXX

We are writing to you concerning one of your patients who recently delivered a baby diagnosed with Congenital Rubella Syndrome (CRS). As cases of CRS potentially remain infectious for upwards of one year and because they, i.e., mother-baby pair, would require ongoing frequent clinical visits, we are advising of best infection control practices aimed at limiting exposure and, ultimately, transmission risk. Please note that the mother in this is no longer infectious.

The following are the recommended best practices:

Booking appointments at the end of the day is preferred.

Preferably, ensure no patients are in the waiting room.

Guide mother and baby directly into a private room.

The recommended precautions for PPE are contact and droplet.

Ensure any staff entering the room mother and baby are in are immune to rubella (either by vaccine or serology) and are not pregnant.

We would like to point out that rubella, being an enveloped virus, can be effectively eliminated by cleaning products meant for low-level disinfection. The Saskatchewan Health Authority currently uses such products, including Accel wipes. For additional IPAC-related information, please review the College of Physicians and Surgeons of Saskatchewan IPAC resource located here https://www.cps.sk.ca/iMIS/Documents/Legislation/Policies/GUIDELINE%20-%20IPAC%20Clinical%20Office%20Practice.pdf

Due to the nature of this case, and to protect the community from local transmission, public health is assisting the mother in transportation to and from appointments. We have a dedicated staff member for this who has other clients they need to assist with throughout the day. It would be greatly appreciated if appointment times could be minimized to reduce potential exposure times to staff and other patients.

Finally, due to organizational risk policy, staff cannot be alone with the baby, watch or care for the infant.

We appreciate your attention to this matter, and please reach out if you have any questions.

Sincerely,

Medical Health Officer
